# Asthma Phenotypes and Host Risk Factors Associated With Various Asthma-Related Outcomes in Health Workers

**DOI:** 10.3389/falgy.2021.747566

**Published:** 2021-10-15

**Authors:** Hussein H. Mwanga, Roslynn Baatjies, Tanusha Singh, Mohamed F. Jeebhay

**Affiliations:** ^1^Division of Occupational Medicine and Center for Environmental & Occupational Health Research, School of Public Health and Family Medicine, University of Cape Town, Cape Town, South Africa; ^2^Department of Environmental and Occupational Health, School of Public Health and Social Sciences, Muhimbili University of Health and Allied Sciences, Dar es Salaam, Tanzania; ^3^Department of Environmental and Occupational Studies, Faculty of Applied Sciences, Cape Peninsula University of Technology, Cape Town, South Africa; ^4^National Institute for Occupational Health, National Health Laboratory Services, Johannesburg, South Africa; ^5^Department of Clinical Microbiology and Infectious Diseases, School of Pathology, University of the Witwatersrand, Johannesburg, South Africa; ^6^Department of Environmental Health, Faculty of Health Sciences, University of Johannesburg, Johannesburg, South Africa

**Keywords:** asthma prevalence, work-related asthma, asthma phenotypes, host risk factors, occupational allergy

## Abstract

**Background:** Work-related asthma phenotypes in health workers (HWs) exposed to cleaning agents have not been investigated extensively as other occupational exposures. This study aimed to describe asthma phenotypes and to identify important host risk factors associated with various asthma-related outcomes.

**Methods:** A cross-sectional study of 699 HWs was conducted in two large tertiary hospitals. A total of 697 HWs completed questionnaire interviews. Sera collected from 682 HWs were analyzed for atopy (Phadiatop) and IgE to occupational allergens (NRL—Hev b5, Hev b6.02; chlorhexidine and ortho-phthalaldehyde—OPA). Methacholine (MCT), bronchodilator challenge (BDR) and fractional exhaled nitric oxide (FeNO) were performed. An asthma symptom score (ASS) used five asthma-related symptoms reported in the past 12 months. Current asthma was based on use of asthma medication or an asthma attack or being woken up by an attack of shortness of breath in the past 12 months. Nonspecific bronchial hyperresponsiveness (NSBH) was defined as having either a positive MCT or a significant bronchodilator response. Two continuous indices of NSBH [continuous index of responsiveness (CIR) and dose-response slope (DRS)] were calculated.

**Results:** The prevalence of current asthma was 10%, atopic asthma (6%) and non-atopic asthma (4%). Overall, 2% of subjects had work-related asthma. There was a weak positive association between NSBH and FeNO [CIR: Beta coefficient (β) = 0.12; CI: 0.03–0.22 and DRS: β = 0.07; CI: 0.03–0.12]. Combining FeNO ≥ 50 ppb with a BDR [mean ratio (MR) = 5.89; CI: 1.02–34.14] or with NSBH (MR = 4.62; CI: 1.16–18.46) correlated better with ASS than FeNO alone (MR = 2.23; CI: 1.30–3.85). HWs with current asthma were twice as likely to be atopic. FeNO was positively associated with atopy (OR = 3.19; CI: 1.59–6.39) but negatively associated with smoking status (GMR = 0.76; CI: 0.62–0.94). Most HWs sensitized to occupational allergens were atopic.

**Conclusion:** Atopic asthma was more prevalent than non-atopic asthma in HWs. Most asthma-related outcomes were positively associated with allergic predictors suggesting a dominant role for IgE mechanisms for work-related symptoms and asthma associated with sensitization to OPA or chlorhexidine.

## Introduction

Asthma is a heterogeneous disease characterized by diverse clinical, physiological and inflammatory attributes (1–3). Various phenotypes for non-work-related asthma have been reported in the literature, with significant efforts directed toward characterizing severe asthma phenotypes (2, 3). However, few studies have investigated work-related asthma phenotypes. Studies that have characterized occupational asthma phenotypes, have focused mainly on the mechanisms (allergic vs. irritant) and etiological agents (high molecular weight—HMW vs. low molecular weight—LMW) (1, 4, 5). Very little is known about work-related asthma phenotypes in health workers (HWs) exposed to cleaning agents (6, 7). Differentiating between various asthma phenotypes in the workplace is important in understanding the heterogeneous nature of the disease to develop appropriate management and preventive strategies (1, 5).

There have been considerable efforts in investigating the burden of asthma among HWs exposed to cleaning agents. In a prospective population-based European study, the prevalence of new-onset asthma (asthma attack or taking asthma medication in the past 12 months) among nurses was reported to be 4.8% (8). Further analysis revealed a slightly higher prevalence of new-onset asthma (currently taking asthma medication, asthma attack or woken up by an attack of shortness of breath in last 12 months) of 6%, most likely due to different asthma definitions used (9). These findings are similar to a US study of HWs (10), which reported an overall prevalence of doctor-diagnosed asthma with onset after entry into the healthcare profession of 6.6%. In this study, nurses (7.3%) had the highest prevalence followed by respiratory therapists (5.6%), occupational therapists (4.5%) and doctors (4.2%). However, a study 2 years later from the same US population reported a much higher prevalence (9.8%) of doctor-diagnosed asthma with onset after entry into the healthcare profession in nurses based on the longest job held (11). Globally, the prevalence of asthma in HWs exposed to cleaning agents ranges between 4.4 and 11.2% (7–13). Very little is known about the burden of asthma among HWs in Africa, except for a study of South African dental HWs (14), in which the prevalence of atopic asthma (6.9%) was higher than non-atopic asthma (5.9%) or work-exacerbated asthma (4%).

Various studies have investigated the host-associated risk factors in adult asthma. Common host risk factors associated with asthma have included age, female sex, seniority, smoking status and atopy (10, 11). A significantly higher risk (RR = 2.9) for new-onset asthma was observed for atopic individuals, those with a parental history of asthma (RR = 2.1) and in non-smokers (RR = 1.8) in the large population-based ECRHS II study of workers across different industries (8). There is inconsistent evidence regarding the association between smoking and asthma in general and occupational asthma in particular (6, 15, 16). Furthermore, very little is known about the risk of smoking in relation to asthma in HWs exposed to cleaning agents (15, 17).

A review on risk factors for non-work-related adult-onset asthma and occupational asthma revealed that host-associated factors (sex, genetics, and atopy) did not differ for these two broad asthma phenotypes, whereas, environmental factors appeared to play a very important role for the occupational asthma phenotype. A later study however identified novel genes implicated in adult asthma related to occupational exposure to LMW agents or irritants (18). Among HWs, women appear to be more affected than men, with the former having a higher prevalence of all asthma phenotypes, including WRA symptoms (3.6 vs. 1.8%), work exacerbated asthma (1.3 vs. 0.3%) and occupational asthma (1.0 vs. 0.1%) than men (19). Similar findings have been reported in the ECRHS II study (8). In the light of these observations, it is likely that the gendered distribution of work plays an important role. Furthermore, female sex hormones have also been implicated in the pathogenesis of asthma and the higher prevalence in women (1).

The aim of this study was to describe asthma phenotypes in health workers exposed to cleaning agents and to identify important host risk factors associated with various asthma-related outcomes, based either on the presence of symptoms, non-specific bronchial hyperresponsiveness or allergic airway inflammation.

## Materials and Methods

### Study Design, Population, and Sampling

A cross-sectional study of 699 HWs was conducted in two large tertiary academic hospitals (346 from a South African hospital—SAH and 353 from the Tanzanian hospital—TAH) during the period July 2014 to March 2015 for the SAH and between September 2017 to March 2018 for the TAH. All permanently employed HWs in high-risk departments using significant amounts of cleaning agents at a frequency much higher than other departments constituted the sampling frame of the study. Study participants were selected from these departments using stratified random sampling according to job title, choosing up to five HWs from each high-risk department. Ethics approval was obtained from the Human Research Ethics Committee (HREC) of the University of Cape Town (HREC Ref: 212/2013), Muhimbili University of Health and Allied Sciences (MUHAS) Institutional Review Board and University of Michigan Medical School Institutional Review Board (HUM00083115).

### Questionnaire

A total of 697 participants completed the questionnaire interviews. Each participant answered a modified questionnaire for the investigation of asthma as contained in the Protocol for the European Community Respiratory Health Survey (ECRHS) (20). The standardized ECRHS questionnaire was originally designed to estimate the prevalence and risk factors for asthma, asthma-like symptoms and airway responsiveness as well as asthma treatment practices in epidemiological studies conducted in the European Community. Asthma-related questions in the ECRHS questionnaire were originally derived from the International Union Against Tuberculosis and Lung Diseases (IUATLD) bronchial symptom questionnaire. The questionnaire used in the current study also included an occupational history section containing validated questions from the National Institute for Occupational Safety and Health (NIOSH) specific questionnaire for cleaning agents in the health care setting (21). The questionnaire was administered by trained interviewers in English for South African health workers (SAHWs) and in Swahili for Tanzanian health workers (TAHWs). The translated Swahili questionnaire was back-translated to ensure validity and repeatability. The questionnaire took approximately 45 minutes to complete.

### Immunological Assessment

Blood samples were collected from 682 participants. Atopy was determined by the Phadiatop test. The quantification of specific IgE antibodies to specific occupational allergens: natural rubber latex—NRL (*Hevea brasiliensis*—Hev b5, Hev b6.02), chlorhexidine and ortho-phthalaldehyde (OPA) used the UniCAP system (Phadia, Uppsala, Sweden) obtained from Thermo Scientific. These particular occupational allergens were studied since HWs complained of work-related asthma and skin symptoms to the nurse manager when working with medical instrument cleaning agents (especially OPA) and chlorhexidine, before the study was initiated. It was also important to study NRL since HWs in the selected hospitals continued to use NRL gloves. In the late 1980s and 1990s, NRL exposure was the major cause of occupational allergy and asthma among health workers in the SAH (22). Further details on the OPA ImmunoCAP are provided elsewhere (23).

### Spirometry and Bronchodilator Challenge (BDR)

There were 328 participants from the TAH and 25 participants from the SAH that performed spirometry and bronchodilator challenge (BDR) tests. Spirometry was conducted according to guidelines of the American Thoracic Society/European Respiratory Society (ATS/ERS) (24) using the EasyOne World spirometer (ndd Medical Technologies, Zurich, Switzerland) in the TAH and the Jaeger Aerosol Provocation System (APS) Pro apparatus in the SAH according to the manufacturer's instructions.

### Methacholine Challenge Tests

Methacholine challenge testing (MCT) was only performed in the South African study site due to logistical and safety considerations. The tests were conducted in a pulmonary function laboratory that was well equipped with appropriate resuscitation facilities. Among 318 participants that underwent spirometry, 239 performed interpretable PD_20_ methacholine results while 52 participants had ≥10% decrease in FEV_1_ after administration of saline diluent and were therefore not considered for MCT. Further details are provided elsewhere (23).

### Fractional Exhaled Nitric Oxide (FeNO)

A total of 654 participants performed FeNO tests. A hand-held portable exhaled nitric oxide sampling device (NIOX MINO® Airway Inflammation Monitor, Aerocrine AB, Solna, Sweden) was used according to the manufacturer's instructions. Two technically adequate measurements were performed in line with the current American Thoracic Society/European Respiratory Society recommendations (25). A third maneuver was performed if the difference between the first two measurements was more than 10 ppb. The FeNO test was done during the work-shift before spirometry / MCT.

### Asthma Diagnosis and Operational Definitions for Asthma Phenotypes

Asthma diagnosis was based on the information obtained from the modified ECRHS questionnaire. Doctor-diagnosed asthma was defined as a positive answer to one of two questions: “have you ever had asthma?” or “have you ever had an asthma attack?”, combined with a positive response to the question: “has your asthma been confirmed by a doctor?” Childhood-onset asthma was defined as doctor-diagnosed asthma at the age of 16 years or younger. Adult-onset asthma was defined as doctor-diagnosed asthma at the age of 17 years or older.

An asthma symptom score (ASS) was computed based on the sum of answers (0=no, 1=yes) to five questions reported in the past 12 months. These included the presence of shortness of breath while wheezing; being woken up with chest tightness; an attack of shortness of breath at rest; an attack of shortness of breath after exercise; and being woken up by an attack of shortness of breath, as has been described in previous studies (12, 26–28). A binary variable was created from these five asthma-related symptoms (≥2 symptoms vs 0–1 symptom). Having ≥ 2 asthma-related symptoms was considered ‘more likely' and 0–1 symptom as ‘less likely' to have asthma.

Information for defining the asthma phenotypes was obtained from the modified ECRHS questionnaire and immunological tests. *Current asthma* was defined as having either an asthma attack, current use of asthma medication or being woken up by an attack of shortness of breath, in the past 12 months (9, 12, 29). *Atopic asthma* and *non-atopic asthma* were differentiated from each other based on Phadiatop test (signifying the presence of atopy) in the presence of the aforementioned definition for current asthma. *Work-related asthma* was based on the presence of work-related chest symptoms in the past 12 months that improves when away from work or worsens on return to work, in the presence of current asthma.

Individuals with sensitization to specific occupational allergens were identified based on sIgE ≥ 0.35 KU/L. A variable was created for sensitization to at least one occupational allergen (OPA, Chlorhexidine or NRL).

A categorical variable for NSBH was defined as fulfilling any of the following two criteria: positive methacholine challenge test (PD_20_ methacholine < 0.4 mg) or significant bronchodilator response (≥12% and ≥ 200 ml increase in FEV_1_ after administration of a bronchodilator).

Two continuous indices of MCT (continuous index of responsiveness: CIR and dose-response slope: DRS) were also calculated (30). CIR = (Post-diluent FEV_1_ – FEV_1_ at the last dose of methacholine) ÷ Post-diluent FEV_1_ and DRS = (Post-diluent FEV_1_ – FEV_1_ at the last dose of methacholine) ÷ (Post-diluent FEV_1_ x Last methacholine dose). CIR and DRS were all multiplied by 100 to convert into percentages.

FeNO results were interpreted as follows: low < 25 ppb; elevated for values 25–50 ppb; and high for values > 50 ppb (31, 32). In addition to FeNO being analyzed as a continuous variable, two categorical variables (FeNO ≥ 25 ppb and FeNO ≥ 50 ppb) were also computed to gain more specificity in the analysis.

### Host-Associated Risk Factors

Further information on the host-associated risk factors was based on information obtained from the questionnaire and immunological tests (for atopy). Body mass index (BMI) was calculated as weight in kilograms divided by height in meters squared. Two categorical variables were created for smoking history. One was a binary variable: ever smokers (current smokers and ex-smokers) vs. never smokers. The second smoking variable was a nominal variable with three categories (current smokers, ex-smokers and never smokers). A family history of allergy was defined as a positive answer to the question “do or did any member of your family (blood relatives) ever have any kind of allergies?”. Individuals with atopy were defined as those subjects having a positive Phadiatop test. Hay-fever was defined as a positive response to the question “have you ever had any nose or eye problems or allergies such as hay fever?”. Frequency of domestic cleaning was categorized as ≥1 day/week vs. <1 day/week. All these variables were obtained from the questionnaire responses.

### Statistical Analysis

All data analysis was performed using statistical software STATA version 14 (StataCorp, College Station, Texas, USA). Numerical variables were summarized using median and interquartile range, since most variables did not follow a normal distribution. Scatter plots, Spearman rank correlational analysis (for non-normally distributed) and linear regression models were used to assess association between numerical variables (CIR, DRS and FeNO). Continuous indices of MCT (CIR and DRS) and FeNO were log-transformed (natural log) before linear regression analysis was conducted.

After conducting univariate and bivariate analyses, unadjusted logistic and linear regression models were developed to test the association between health outcomes (asthma-related symptoms, allergic sensitization to occupational allergens, NSBH, FeNO, current asthma, atopic asthma, non-atopic asthma, work-related asthma) and host-related risk factors (age, gender, BMI, atopy, smoking history, medical history and domestic chemical exposures). A negative binomial regression analysis was used for exploring the association between ASS (a count outcome variable) and host-related risk factors. The results of negative binomial regression models were reported as mean ratios with 95% confidence intervals. A *p* < 0.05 was regarded as statistically significant.

## Results

### Asthma Phenotypes

The overall prevalence of current asthma was 10%, with atopic asthma (6%) slightly more prevalent than non-atopic asthma (4%) (Table 1). There were 2% of subjects with work-related asthma.

**Table 1:** Prevalence of asthma-related outcomes in health workers working with cleaning agents.

	**Prevalence** ***N* (%)**
**Participants (*n*)**	**697**
**Asthma symptom score**	
0	478 (69)
1	128 (18)
2	42 (6)
3	17 (2)
4	18 (3)
5	14 (2)
**Asthma history**	
Ever asthma	52 (7)
Ever asthma attack	55 (8)
Doctor-diagnosed asthma	48 (7)
Current use of asthma medication	39 (6)
Asthma attack in the past 12 months	31 (5)
Woken up by an attack of shortness of breath in the last 12 months	48 (7)
Current use of asthma medication OR asthma attack in the past 12 months	44 (6)
**Work-related symptoms**	
Work-related chest symptoms in the past 12 months	48 (7)
Work-related ocular–nasal symptoms in the past 12 months	109 (16)
**Atopy** (*n =* 682)	296 (43)
**Bronchial hyperresponsiveness**	
Positive Bronchodilator response (BDR): ≥12% and ≥200 ml FEV_1_ increase post-bronchodilator (*n =* 207)	26 (13)
Methacholine challenge test: PD_20_ methacholine <0.4 mg (*n =* 239)	31 (13)
NSBH^#^ (*n =* 446)	57 (13)
**Fractional exhaled nitric oxide (FeNO)** (*n =* 654)	
FeNO (ppb) [median (IQR)]	17 (11–24)
≥25 ppb	150 (23)
≥50 ppb	41 (6)
**Asthma phenotypes**	
Current asthma	69 (10)
Atopic asthma	41 (6)
Non-atopic asthma	28 (4)
Work-related asthma	17 (2)

*FEV_1_, forced expiratory volume in 1 s; PD_20_ methacholine, provocative dose of methacholine causing a ≥ 20% fall in FEV_1_; NSBH, non-specific bronchial hyperresponsiveness*;

# *NSBH defined as any of the following two criteria: PD_20_ methacholine <0.4 mg OR a positive BDR defined as ≥12% and ≥200 ml increase in FEV_1_ after administration of a bronchodilator; IQR, interquartile range*.

### Association Between FeNO and/or NSBH and Asthma Symptoms

There was a statistically significant weak positive correlation between continuous indices of non-specific bronchial hyperresponsiveness (CIR: β = 0.12 and DRS: β = 0.07) and FeNO (Table 2). A similar pattern was observed between NSBH [CIR: Odds ratio (OR) = 1.65 and DRS: OR = 1.25] and FeNO ≥ 25 ppb. However, only 3–5% of the variability in NSBH could be explained by the FeNO levels. Further analysis found that levels of FeNO ≥ 50 ppb combined with either a positive BDR [mean ratio (MR) = 5.89] or increased NSBH (MR = 4.62) was more strongly associated with asthma symptoms than FeNO levels on its own (FeNO ≥ 50 ppb: MR = 2.23) (Table 3). Furthermore, an ASS ≥2 appeared to be a better discriminator of asthma than the presence of any one asthma symptom. Therefore, ASS ≥2 formed the basis for further investigation with host factors.

**Table 2:** Association between continuous indices of bronchial hyperresponsiveness obtained from methacholine challenge and exhaled nitric oxide (FeNO).

	**FeNO**			
	**FeNO, ppb** ^**#**^		**FeNO ≥25 ppb**	**FeNO ≥50 ppb**
	**Beta coefficient (95% CI)**	***R* ^2^**	**Odds ratio (95% CI)**	
Continuous index of responsiveness (CIR)^#^	0.12 (0.03–0.22)^*^	0.03	1.65 (1.10–2.47)^*^	1.69 (0.88–3.27)
Dose-response slope (DRS)^#^	0.07 (0.03–0.12)^**^	0.05	1.25 (1.06–1.47)^**^	1.18 (0.93–1.50)

*Each odds ratio/Beta coefficient represents a separate unadjusted regression model; CI, Confidence Interval*;

# *natural log-transformed values*;

* *p < 0.05*;

** *p < 0.01*.

**Table 3:** Association between FeNO and/or bronchial hyperresponsiveness and asthma symptoms in health workers working with cleaning agents.

	**Prevalence: *n* (%)**	**Asthma symptoms**	
		**Asthma symptom score**	**Asthma symptom score (≥2 vs. 0–1)**
		**MR (95% CI)**	**OR (95% CI)**
Prevalence: *n* (%)			91 (13)
FeNO ≥ 25 ppb (*n =* 654)	150 (23)	1.53 (1.09–2.16)^*^	1.61 (0.97–2.66)
FeNO ≥ 50 ppb (*n =* 654)	39 (6)	2.23 (1.30–3.85)^**^	3.10 (1.51–6.34)^**^
Positive BDR (*n =* 207) ^¶^	26 (13)	0.87 (0.27–2.78)	0.55 (0.12–2.46)
PD_20_M <0.4 mg (*n =* 239)*^#^*	31 (13)	1.26 (0.76–2.08)	1.54 (0.62–3.82)
NSBH (*n =* 446)	57 (13)	1.17 (0.71–1.94)	1.16 (0.54–2.50)
FeNO and/or positive BDR (*n* = 662)			
FeNO ≥ 25 ppb OR positive BDR	168 (25)	1.31 (0.94–1.83)	1.32 (0.80–2.17)
FeNO ≥ 25 ppb AND positive BDR	8 (1)	2.42 (0.74–7.90)	2.25 (0.45–11.35)
FeNO ≥ 50 ppb OR positive BDR	62 (9)	1.45 (0.89–2.36)	1.70 (0.87–3.34)
FeNO ≥ 50 ppb AND positive BDR	3 (1)	5.89 (1.02–34.14)^*^	13.64 (1.22–152.11)^*^
FeNO and/or PD_20_M <0.4 mg^#^(*n =* 337)			
FeNO ≥ 25 ppb OR PD_20_M <0.4 mg	103 (31)	1.25 (0.90–1.75)	1.31 (0.72–2.40)
FeNO ≥ 25 ppb AND PD_20_M <0.4 mg	11 (3)	1.47 (0.66–3.29)	1.92 (0.49–7.47)
FeNO ≥ 50 ppb OR PD_20_M <0.4 mg	51 (15)	1.52 (1.01–2.28)^*^	2.18 (1.09–4.38)^*^
FeNO ≥ 50 ppb AND PD_20_M <0.4 mg	2 (1)	2.01 (0.36–11.20)	5.06 (0.31–82.04)
FeNO and/or NSBH (*n* = 665)			
FeNO ≥ 25 ppb OR NSBH	188 (28)	1.38 (1.00–1.90)	1.42 (0.88–2.30)
FeNO ≥ 25 ppb AND NSBH	19 (3)	2.17 (0.99–4.75)	2.48 (0.87–7.08)
FeNO ≥ 50 ppb OR NSBH	91 (14)	1.56 (1.04–2.34)^*^	1.83 (1.03–3.25)^*^
FeNO ≥ 50 ppb AND NSBH	5 (1)	4.62 (1.16–18.46)^*^	10.39 (1.71–63.11)^*^

# *South African HCWs only*;

¶ *≥12% and ≥200 ml FEV_1_ increase post-bronchodilator; BDR, Bronchodilator response; MR, mean ratio; OR, odds ratio; CI, confidence interval; FEV_1_, forced expiratory volume in 1 s; PD_20_M, provocative dose of methacholine causing a ≥20% fall in FEV1; NSBH, non-specific bronchial hyperresponsiveness; NSBH defined as any of the following two criteria: PD_20_M <0.4 mg OR positive BDR: ≥12% and ≥200 ml increase in FEV1 after administration of a bronchodilator*;

* *p < 0.05*;

** *p < 0.01; Each OR represents a separate unadjusted regression model*.

### Host Risk Factors Associated With Respiratory Symptoms

In the exploration of demographic host risk factors (Table 4), higher mean asthma symptom scores were more likely to be found in women (mean ratio (MR) = 2.03), those with a higher BMI (MR = 1.04) and atopic individuals (MR = 1.74). Furthermore, a family history of allergy, hay fever, chronic bronchitis or pulmonary tuberculosis (PTB) were also significant predictors. Participants with ASS ≥ 2 were more likely to have ever smoked (OR = 1.99; 95% CI: 1.13–3.49).

**Table 4:** Host risk factors associated with respiratory symptoms among health workers working with cleaning agents.

	**Prevalence: *n* (%)**	**Asthma symptom score^##^**	**Asthma symptom score** **(≥2 vs. 0–1)**	**WRONS**	**WRAS**
Prevalence: *n* (%) (*n =* 697)			91 (13)	109 (16)	48 (7)
**Demographic characteristics**					
Age		1.01 (1.00–1.03)	1.02 (1.00–1.04)^*^	0.99 (0.97- 1.01)	1.00 (0.97–1.03)
Gender (Females vs. Males):		2.03 (1.37–3.00)^***^	1.98 (1.05–3.74)^*^	1.12 (0.68–1.86)	3.25 (1.15–9.19)^*^
BMI		1.04 (1.02–1.06)^**^	1.04 (1.01–1.08)^**^	0.99 (0.96–1.02)	1.00 (0.96–1.04)
**Smoking history**					
Smoking (ever) ^‡‡^	90 (13)	1.48 (0.99–2.23)	1.99 (1.13–3.49)^*^	1.30 (0.74–2.31)	0.60 (0.21–1.70)
Current smoking	42 (6)	1.48 (0.83–2.63)	1.75 (0.78–3.92)	1.75 (0.83–3.68)	0.31 (0.04–2.32)
**Allergy history**					
Family history of allergy	353 (51)	2.11 (1.58–2.82)^***^	2.60 (1.61–4.19)^***^	1.13 (0.75–1.70)	1.16 (0.65–2.10)
Atopy (positive Phadiatop) (*n =* 682)	296 (43)	1.74 (1.30–2.33)^***^	2.40 (1.52–3.79)^***^	1.33 (0.88–2.02)	1.67 (0.92–3.02)
**Medical history**					
Hay fever	306 (44)	2.61 (1.97–3.47)^***^	4.03 (2.47–6.56)^***^	8.16 (4.84–13.76)^***^	3.03 (1.61–5.69)^**^
Childhood-onset (≤ 16 yrs) asthma	19 (3)	4.35 (2.17–8.74)^***^	13.00 (4.97–33.99)^***^	0.63 (0.14–2.76)	2.64 (0.74–9.39)
Adult-onset (>16 yrs) asthma	29 (4)	4.57 (2.63–7.93)^***^	9.73 (4.51–21.02)^***^	1.77 (0.74–4.24)	10.42 (4.59–23.67)^***^
Repeated childhood chest infections	76 (11)	1.53 (0.99–2.37)	1.76 (0.96–3.26)	1.66 (0.93–2.97)	1.71 (0.77–3.80)
Chronic bronchitis	44 (6)	2.79 (1.69–4.60)^***^	3.49 (1.77–6.87)^***^	1.89 (0.92–3.86)	2.30 (0.92–5.74)
Pulmonary tuberculosis	32 (5)	2.21 (1.21–4.06)^*^	4.45 (2.10–9.45)^***^	1.26 (0.51–3.14)	4.26 (1.74–10.44)^**^
**Domestic chemical exposures**					
Home cleaning in the past 12 months (≥1 day vs. <1 day/week)	601 (86)	1.54 (0.98–2.44)	1.76 (0.82–3.77)	0.92 (0.51–1.64)	1.40 (0.54–3.64)
Use of sprays for home cleaning in the past 12 months (≥1 day vs. <1 day/week)	218 (31)	1.47 (1.08–1.99)^*^	1.37 (0.87–2.16)	0.90 (0.57–1.41)	0.81 (0.42–1.55)

*Data are presented as OR (95% CI), unless otherwise indicated*.

## *MR (95% CI); OR, odds ratio; CI, confidence interval; MR, mean ratio*;

* *p < 0.05*;

** *p < 0.01*;

*** *p < 0.001*;

‡‡ *current and ex-smokers vs. never smokers; WRONS: Work-related ocular–nasal symptoms (ocular–nasal symptoms experienced at work in the past 12 months that gets better when away from work OR worsen on return to work; WRAS, Work-related asthma symptoms (asthma symptoms experienced at work in the past 12 months that gets better when away from work OR worsen on return to work; Each OR represents a separate unadjusted regression model*.

Workers with work-related ocular-nasal symptoms (WRONS) had an eight-fold increased odds of a past history of hay fever. Participants with work-related asthma symptoms (WRAS) were more likely to be women (OR = 3.25), have a previous history of hay fever (OR = 3.03) or PTB (OR = 4.26) and were also more likely to report adult-onset asthma (OR = 10.42).

### Host Risk Factors Associated With Allergic Sensitization to Occupational Allergens

Most study participants that were sensitized to either one of the occupational allergens (OPA, chlorhexidine and NRL) were atopic (*p* < 0.001), indicating a very strong association with atopy. Those sensitized to OPA were more likely to have a lower BMI (OR = 0.92) (Table 5). HWs sensitized to at least one occupational allergen (OPA, NRL or chlorhexidine) had a two-fold increased odds of having hay fever.

**Table 5:** Host risk factors associated with allergic sensitization to occupational allergens among health working with cleaning agents.

	**Prevalence: *n* (%)**	**Sensitization to OPA**	**Sensitization to at least one occupational allergen**
Prevalence: *n* (%) (*n =* 682)		26 (4)	34 (5)
**Demographic characteristics**			
Age		0.98 (0.94–1.01)	0.98 (0.95–1.02)
Gender (Females vs. Males)		1.53 (0.52–4.52)	1.06 (0.45–2.49)
BMI		0.92 (0.86–0.99)^*^	0.95 (0.89–1.00)
**Smoking history**			
Smoking (ever) ^‡‡^	90 (13)	0.26 (0.04–1.98)	0.90 (0.31–2.63)
Current smoking	42 (6)	0.57 (0.08–4.30)	1.48 (0.43–5.08)
**Allergy history**			
Family history of allergy	353 (51)	1.36 (0.61 −3.00)	1.26 (0.63–2.52)
Atopy (positive Phadiatop)	296 (43)	NC^***^	NC^***^
**Medical history**			
Hay fever	306 (44)	2.05 (0.92–4.58)	2.09 (1.03–4.24)^*^
Childhood-onset (≤ 16 yrs) asthma	19 (3)	NC	NC
Adult-onset (>16 yrs) asthma	29 (4)	3.15 (0.89–11.17)	2.31 (0.66–8.04)
Repeated childhood chest infections	76 (11)	1.05 (0.31–3.60)	0.77 (0.23–2.59)
Chronic bronchitis	44 (6)	NC	0.90 (0.21–3.88)
Pulmonary tuberculosis	32 (5)	0.80 (0.11–6.13)	1.28 (0.29–5.61)
**Domestic chemical exposures**			
Home cleaning in the past 12 months(≥1 day vs. <1 day/week)	601 (86)	0.89 (0.30–2.64)	0.94 (0.35–2.49)
Use of sprays for home cleaning in the past 12 months (≥1 day vs. <1 day/week)	218 (31)	0.80 (0.33–1.92)	1.04 (0.50–2.18)

*Data presented as OR (95% CI); OR, odds ratio; CI, confidence interval*;

* *p < 0.05*;

*** *p < 0.001*;

‡‡ *: current & ex-smokers vs. never smokers; NC, not calculable; Each OR represents a separate unadjusted logistic regression model*.

### Host Risk Factors Associated With Non-specific Bronchial Hyperresponsiveness and Airway Inflammation

Non-specific bronchial hyperresponsiveness (as evidenced by increased DRS) was strongly associated with being female [Geometric mean ratio (GM ratio) = 3.40], being atopic (GM ratio = 1.83), having a history of hay fever (GM ratio = 2.01), childhood-onset asthma (GM ratio = 9.01) or chronic bronchitis (GM ratio = 2.61) (Table 6).

**Table 6:** Host risk factors associated with bronchial hyperresponsiveness and airway inflammation among health workers working with cleaning agents.

	**Prevalence: *n* (%)**	**Dose-response slope (DRS)^**‡**^** **(*n =* 239)**	**NSBH** **(*n =* 446)**	**FeNO, ppb^**‡**^** **(*n =* 654)**	**FeNO♯ 50 ppb** **(*n =* 654)**
Prevalence: n (%)			57 (13)		41 (6)
**Demographic characteristics**					
Age		1.00 (0.98–1.03)	1.02 (0.99–1.05)	1.00 (1.00–1.01)	0.99 (0.96–1.02)
Gender (Females vs. Males)		3.40 (1.72–6.73)^***^	2.08 (0.91–4.75)	0.87 (0.77–0.98)^*^	0.84 (0.40–1.76)
BMI		0.98 (0.96–1.01)	1.00 (0.97–1.03)	1.00 (1.00–1.01)	1.01 (0.98–1.04)
**Smoking history**					
Smoking (ever) ^‡‡^	90 (13)	0.88 (0.48–1.61)	1.07 (0.50–2.29)	0.88 (0.76–1.02)	0.16 (0.02–1.16)
Current smoking	42 (6)	1.02 (0.46–2.26)	0.98 (0.33–2.92)	0.76 (0.62–0.94)^**^	0.34 (0.05–2.52)
**Allergy history**					
Family history of allergy	353 (51)	1.25 (0.72–2.18)	1.01 (0.58–1.77)	1.04 (0.94–1.15)	1.21 (0.64–2.29)
Atopy (positive Phadiatop) (*n =* 682)	296 (43)	1.83 (1.08–3.10)^*^	1.22 (0.70–2.12)	1.38 (1.25–1.52)^***^	3.19 (1.59–6.39)^**^
**Medical history**					
Hay fever	306 (44)	2.01 (1.19–3.39)^**^	1.16 (0.67–2.03)	1.16 (1.05–1.28)^**^	2.05 (1.08–3.93)^*^
Childhood-onset (≤ 16 yrs) asthma	19 (3)	9.01 (1.59–50.93)^*^	1.74 (0.48–6.37)	1.49 (1.11–1.99)^**^	2.94 (0.82–10.52)
Adult-onset (>16 yrs) asthma	29 (4)	2.91 (0.88–9.61)	0.71 (0.16–3.12)	1.22 (0.95–1.57)	1.93 (0.56–6.70)
Repeated childhood chest infections	76 (11)	1.31 (0.49–3.49)	0.56 (0.19–1.62)	1.03 (0.87–1.20)	1.17 (0.44–3.08)
Chronic bronchitis	44 (6)	2.61 (1.20–5.67)^*^	2.41 (1.07–5.42)^*^	1.19 (0.98–1.46)	1.13 (0.33–3.81)
Pulmonary tuberculosis	32 (5)	0.46 (0.14–1.44)	0.35 (0.05–2.64)	1.10 (0.87–1.39)	1.03 (0.24–4.47)
**Domestic chemical exposures**					
Home cleaning in the past 12 months (≥1 day vs. <1 day/week)	601 (86)	3.12 (0.84–11.62)	1.83 (0.63–5.28)	0.95 (0.82–1.09)	1.16 (0.44–3.05)
Use of sprays for home cleaning in the past 12 months (≥1 day vs. <1 day/week)	218 (31)	1.28 (0.74–2.21)	1.71 (0.98–2.99)	1.13 (1.01–1.25)^*^	1.09 (0.56–2.12)

*Data presented as OR (95% CI), unless otherwise indicated; OR, odds ratio; CI, confidence interval*;

‡ *Geometric mean ratio (95% CI)*;

* *p < 0.05*;

** *p < 0.01*;

*** *p < 0.001*;

‡‡ *current & ex-smokers vs. never smokers; NSBH: non-specific bronchial hyperresponsiveness; NSBH defined as any of the following two criteria: PD_20_ methacholine <0.4 mg OR positive BDR: ≥12% and ≥ 200 ml increase in FEV_1_ after administration of a bronchodilator; Each OR represents a separate unadjusted regression model*.

For airway inflammation, atopy (GM ratio = 1.38), history of hay fever (GM ratio = 1.16) or childhood-onset asthma (GM ratio = 1.49) were significantly associated with increased FeNO. On the other hand, current smoking (GM ratio = 0.76) and being female (GM ratio = 0.87) were negatively associated with FeNO. Furthermore, those with FeNO ≥ 50 ppb were more likely to be atopic (OR = 3.19) or have a history of hay fever (OR = 2.05).

### Host Risk Factors Associated With Asthma Phenotypes

Health workers with current asthma were more likely to be atopic (OR = 2.04), have a family history of allergy (OR = 2.25), history of hay fever (OR = 2.30) or childhood lung disease (Table 7). Similarly, atopic asthma was positively associated with a family history of allergy, hay fever, repeated childhood chest infections, chronic bronchitis and skin symptoms, whereas those with non-atopic asthma were more likely to be current smokers or have a past history of PTB. Work-related asthma on the other hand, was positively associated with atopy (OR = 3.20; 95% CI: 1.12–9.19), a history of hay fever (OR = 3.15; 95% CI: 1.10–9.04) and having had PTB (OR = 10.08; 95% CI: 3.31–30.64). Of note, exposure to cleaning agents in the domestic setting was not associated with most of the asthma indices investigated.

**Table 7:** Host risk factors associated with asthma phenotypes among health workers working with cleaning agents.

	**Prevalence: *n* (%)**	**Current asthma**	**Atopic Asthma**	**Non-atopic Asthma**	**Work-related Asthma**
Prevalence: *n* (%) (*n =* 697)		69 (10)	41 (6)	28 (4)	17 (2)
**Demographic characteristics**					
Age		1.03 (1.01–1.06)^*^	1.02 (0.99–1.05)	1.04 (1.00–1.08)^*^	1.01 (0.97–1.06)
Gender (females vs. males)		1.72 (0.86–3.46)	2.68 (0.94–7.65)	1.02 (0.41–2.56)	NC
BMI		1.02 (1.00–1.05)	1.03 (1.00–1.06)	1.01 (0.96–1.05)	1.03 (0.99–1.06)
**Smoking history**					
Smoking (ever) ^‡‡^	90 (13)	1.32 (0.66–2.62)	0.72 (0.25–2.06)	2.35 (0.97–5.71)	0.42 (0.05–3.17)
Current smoking	42 (6)	1.89 (0.81–4.45)	0.77 (0.18–3.31)	3.77 (1.35–10.57)^*^	0.90 (0.12–6.96)
**Allergy history**					
Family history of allergy	353 (51)	2.25 (1.32–3.82)^**^	2.48 (1.24–4.94)^*^	1.80 (0.82–3.95)	1.40 (0.53–3.73)
Atopy (positive Phadiatop) (*n =* 682)	296 (43)	2.04 (1.23–3.39)^**^	NA	NA	3.20 (1.12–9.19)^*^
**Medical history**					
Hay fever	306 (44)	2.30 (1.38–3.83)^**^	2.93 (1.49–5.76)^**^	1.50 (0.70–3.20)	3.15 (1.10–9.04)^*^
Childhood-onset (≤ 16 yrs) asthma	19 (3)	31.72 (11.01–91.33)^***^	29.70 (11.13–79.26)^***^	4.90 (1.34–17.90)^*^	8.89 (2.32–34.03)^**^
Adult-onset (>16 yrs) asthma	29 (4)	51.83 (20.11–133.63)^***^	15.55 (6.80–35.58)^***^	23.40 (9.60–57.06)^***^	37.13 (12.98–106.21)^***^
Repeated childhood chest infections	76 (11)	2.08 (1.08–4.02)^*^	2.47 (1.13–5.40)^*^	1.38 (0.47–4.10)	1.78 (0.50–6.35)
Chronic bronchitis	44 (6)	3.42 (1.64–7.12)^**^	3.44 (1.43–8.29)^**^	2.62 (0.87–7.92)	2.03 (0.45–9.15)
Pulmonary tuberculosis	32 (5)	3.95 (1.75–8.91)^**^	1.71 (0.50–5.86)	6.75 (2.52–18.05)^***^	10.08 (3.31–30.64)^***^
**Skin symptoms**					
Skin symptoms (ever)	215 (31)	1.51 (0.90–2.51)	2.25 (1.19–4.25)^*^	0.74 (0.31–1.77)	0.93 (0.32–2.68)
Two or more episodes of skin symptoms in the last 12 months	125 (18)	1.31 (0.71–2.40)	1.52 (0.72–3.18)	1.00 (0.37–2.67)	1.42 (0.46–4.44)
Symptoms affecting hands or forearms^#^	86 (12)	1.40 (0.70–2.78)	1.50 (0.65–3.51)	1.19 (0.40–3.53)	1.54 (0.43–5.48)
Symptoms affecting the whole body^#^	36 (5)	0.82 (0.25–2.75)	1.49 (0.44–5.08)	NC	2.53 (0.56–11.53)
Work-related skin symptoms (ever)	130 (19)	1.49 (0.83–2.67)	1.89 (0.94–3.81)	0.95 (0.35–2.54)	0.93 (0.26–3.30)
Work-related skin symptoms in the past 12 months	80 (12)	1.18 (0.56–2.47)	1.35 (0.55–3.31)	0.92 (0.27–3.13)	0.48 (0.06–3.63)
Doctor-diagnosed work-related skin symptoms (ever)	20 (13)	1.63 (0.47–5.72)	2.97 (0.83–10.57)	NC	2.17 (0.27–17.25)
**Domestic chemical exposures**					
Home cleaning in the past 12 months (≥1 day vs. <1 day/week)	601 (86)	0.94 (0.46–1.90)	1.51 (0.53–4.33)	0.57 (0.23–1.44)	1.20 (0.27–5.35)
Use of sprays for home cleaning in the past 12 months(≥1 day vs. <1 day/week)	218 (31)	1.11 (0.66–1.88)	1.44 (0.75–2.75)	0.72 (0.30–1.73)	0.91 (0.32–2.63)

*Data presented as OR (95% CI); OR, odds ratio; CI, confidence interval*;

* *p < 0.05*;

** *p < 0.01*;

*** *p < 0.001*;

‡‡ *: current & ex-smokers vs. never smokers*;

# *presence of itchy/scratchy skin, hives, dry/scaly skin, redness of the skin, blisters/weeping skin or burning skin; NC, not calculable; NA, not applicable; Each OR represents a separate unadjusted logistic regression model*.

## Discussion

In this study of health workers employed in two large tertiary academic hospitals in southern Africa, atopic asthma (6%) was more prevalent than non-atopic asthma (4%). Furthermore, most asthma-related outcomes and work-related symptoms were positively associated with host attributes suggesting a more dominant role for allergic mechanisms underlying asthma and work-related symptoms. More stronger associations were also observed for asthma symptoms when FeNO ≥ 50 ppb (a marker of allergic airway inflammation) was combined with NSBH (BDR or MCT positive). However, the weak positive association between NSBH and FeNO suggests that these two outcomes possibly detect different underlying pathophysiological mechanisms producing different asthma phenotypes. The pathophysiological mechanisms in asthma related to cleaning agents have not been fully elucidated for most agents. Previous studies have suggested the involvement of both allergic and irritant mechanisms although the latter has been generally thought to be the dominant mechanism (6, 33). Furthermore, it is probable that both these mechanisms may enhance each other, in that airway epithelial damage due to irritant exposures can also activate an allergic T helper type 2 (Th2) response and increase the risk of sensitization (6, 33).

The prevalence of current asthma (10%) in this study was similar to a Spanish study (11%) of professional cleaners (including hospital cleaners) (12) but higher than that reported for nurses (6%) in a general population study of 13 European countries (9). The definition of current asthma used in the current study was similar to these two European studies (9, 12). The reported prevalence of asthma among HWs has varied across other studies as has been reported for other occupational groups probably due to differences in asthma definitions used (34). However, in this current study, the prevalence of atopic asthma (6%) was similar to that reported for South African dental HWs (6.9%) and other workplace-based studies of occupational groups (5–6%) (31–33). On the other hand, the prevalence of non-atopic asthma was slightly lower (4%) than for dental HWs (5.9%) and the other occupational groups (6-7%)(1, 14, 32, 33). Furthermore, the 2% prevalence of work-related asthma is on the lower end of the spectrum of prevalence (3–13%) reported from workplace based studies of other occupational groups in South Africa exposed primarily to high molecular weight agents (14, 35, 36).

In this current study, strong associations were observed between sensitization to occupational allergens in health care settings and atopic status. With exception of one non-atopic subject sensitized to OPA, all HWs sensitized to occupational allergens (OPA, chlorhexidine, and NRL) were atopic (*p* < 0.001). While most studies globally and several South African studies have shown a higher likelihood of atopics being sensitized to high molecular weight agents such as natural rubber latex, species, flour and poultry dust (35–38), this association has not been previously observed for low molecular weight agents such as OPA and chlorhexidine. However, serum specific IgE to OPA and chlorhexidine has been detected in a few studies (39–42). In addition, case reports of asthma due to OPA have also reported a latency period between initial exposure to OPA and the development of symptoms implicating an allergic response caused by OPA (43, 44). Furthermore, animal studies have also suggested that OPA is a respiratory and dermal sensitizer as evidenced by a predominant expression of Th2 cytokines (IL-4, IL-5, and IL-13) in OPA exposed mice (45, 46). Overall, this suggests that the underlying mechanism of asthma in these HWs is probably IgE-mediated. Interestingly, animal studies have also suggested that OPA also has more irritant effects than GTA in both *in-vitro* and *in-vivo* tests, so this irritant effect cannot be ruled out completely (45). Future studies are needed to further investigate these effects to obtain a better understanding of the allergic and possibly irritant pathophysiological mechanisms of asthma due to these cleaning agents.

As to be expected, FeNO, generally considered to be a non-invasive marker for eosinophilic airway inflammation, was positively associated with allergic predictors (atopy and history of hay fever) and a history of childhood-onset asthma. Similarly, stronger associations were also observed between asthma symptoms and high FeNO (≥50 ppb) levels. A positive association between FeNO and atopy is well-known and has also been reported in various studies in other settings (47). Furthermore, a positive association [OR_unadj_ = 3.59, CI: 1.63–7.93; OR_adj_ (atopy + smoking) = 1.93, CI: 0.85–4.37] was also observed between elevated FeNO (≥25 ppb) and allergic sensitization to OPA/chlorhexidine (data not shown in tables). However, much stronger relationships have been demonstrated in workplace-based studies of South African workers exposed to predominantly high molecular weight agents (37, 48). Predictably, current smoking (GM ratio = 0.76) was negatively associated with FeNO levels in HWs as has been observed in other studies (31, 44).

In this current study, a positive association was observed between female gender and asthma-related outcomes including work-related asthma symptoms and bronchial hyperresponsiveness. New-onset asthma in adulthood has been shown to be more prevalent in women, due to multiple factors including female sex hormones being implicated (1). Furthermore, the high preponderance of women workers in the health care setting, as in the current study in which 78% were women, together with the gendered distribution of work is another contributory factor. Overall, the findings of this study are consistent with other studies of asthma associated with cleaning agents, which have reported an increased risk in women since they are more likely to be exposed to cleaning agents than men in their work (19, 49, 50).

Interestingly, in the current study, a past history of pulmonary tuberculosis (TB) was strongly associated with asthma symptoms, current asthma and work-related asthma. This association persisted even after adjusting for age, gender, smoking, atopy and body mass index. Similar findings have been reported in other South African population-based and workplace-based studies (51). In a study of adults exposed to vapors emanating from a sulfur stockpile fire incident, a previous history of TB (more than 1 year prior to the fire) was an important predictor for chronicity of new onset irritant-induced asthma (52). Due to the cross-sectional nature of the current study, it was not possible to establish a temporal relationship between TB and asthma-related outcomes. Furthermore, it is also possible that the observed association between TB and asthma-related outcomes could be due to common post-TB complications such as chronic obstructive pulmonary disease and bronchiectasis (53).This observation needs further exploration in a larger longitudinal study of these health workers.

In this study, smoking was positively associated with an elevated ASS (≥2 vs. 0–1) as well as with non-atopic asthma. However, smoking was not associated with allergic sensitization due to occupational allergens, NSBH, current asthma, work-related ocular-nasal symptoms, nor with work-related asthma. This is consistent with the current body of evidence of asthma on the lack of association between smoking and occupational asthma (1, 6, 15). While some studies have demonstrated that smoking at baseline increases the risk of incident asthma in adulthood, no significant associations have been reported in a cross-sectional analysis at follow-up (16). Overall, various studies have reported inconsistent findings of the association between smoking and asthma in general and occupational asthma in particular (1, 15). Furthermore, very limited specific information is available on the risk of smoking in relation to asthma in HWs exposed to cleaning agents (15, 17). Zock et al. study of cleaning workers also did not demonstrate any association between smoking and asthma (17). Additional longitudinal studies are needed to better understand the relationship between smoking, occupational allergic sensitization and asthma risk among health workers exposed to cleaning agents.

Despite the major strengths of this study in using multiple objective markers for asthma, it is likely that some limitations could have been present in a study of this nature, since it was a cross-sectional study. Furthermore, some of the asthma indices such as current asthma, ASS and work-related asthma were based on self-reported information from questionnaires. Self-reported symptom information is usually characterized by a high sensitivity but low specificity in identifying individuals with asthma. However, since self-reported asthma information obtained from a standardized questionnaire correlated relatively well with NSBH and FeNO in this study, this suggests that it did not impact significantly on the associations observed, although the temporality could not be ascertained with certainty.

In conclusion, this study has demonstrated that atopic asthma was more prevalent than non-atopic asthma in health workers. Furthermore, most asthma-related outcomes and work-related symptoms were positively associated with allergic attributes suggesting a more dominant role of allergic mechanisms over non-allergic mechanisms. That atopic individuals were more likely to be sensitized to LMW agents such as OPA and chlorhexidine, further suggests an IgE mediated mechanism, probably underlying the asthma reported in this group of HWs. Further studies are needed to confirm the findings that have been reported in this study, which to our knowledge have not been previously reported.

## Data Availability Statement

The raw data supporting the conclusions of this article will be made available by the authors, without undue reservation.

## Ethics Statement

The studies involving human participants were reviewed and approved by Human Research Ethics Committee (HREC) of the University of Cape Town (HREC Ref: 212/2013), Muhimbili University of Health and Allied Sciences (MUHAS) Institutional Review Board and University of Michigan Medical School Institutional Review Board (HUM00083115). The participants provided their written informed consent to participate in this study.

## Author Contributions

HM and MJ were responsible for the conceptualization of the study. HM and RB were responsible for the fieldwork. TS conducted the laboratory analysis of the serum samples. HM was responsible for data collection, data management, analysis, and write-up under the supervision of RB and MJ. HM was responsible for preparation of the first draft of the manuscript. All authors contributed to and reviewed the manuscript before submission.

## Funding

This work was supported by the South African Medical Research Council, Millennium Promise Programme NIH Grant 1D43ES018744-01 (University of Michigan/Fogarty International Center), Allergy Society of South Africa & National Research Foundation of South Africa.

## Conflict of Interest

The authors declare that the research was conducted in the absence of any commercial or financial relationships that could be construed as a potential conflict of interest.

## Publisher's Note

All claims expressed in this article are solely those of the authors and do not necessarily represent those of their affiliated organizations, or those of the publisher, the editors and the reviewers. Any product that may be evaluated in this article, or claim that may be made by its manufacturer, is not guaranteed or endorsed by the publisher.
